# Genome Sequences of Multidrug-Resistant, Colistin-Susceptible and -Resistant *Klebsiella pneumoniae* Clinical Isolates from Pakistan

**DOI:** 10.1128/genomeA.01419-16

**Published:** 2016-12-15

**Authors:** Matthew A. Crawford, Ruth Timme, Sara Lomonaco, Christine Lascols, Debra J. Fisher, Shashi K. Sharma, Errol Strain, Marc W. Allard, Eric W. Brown, Melinda A. McFarland, Tim Croley, Thomas S. Hammack, Linda M. Weigel, Kevin Anderson, David R. Hodge, Segaran P. Pillai, Stephen A. Morse, Erum Khan, Molly A. Hughes

**Affiliations:** a Division of Infectious Diseases & International Health, Department of Medicine, University of Virginia, Charlottesville, Virginia, USA; b Center for Food Safety & Applied Nutrition, U.S. Food & Drug Administration, College Park, Maryland, USA; c National Center for Emerging and Zoonotic Diseases, Centers for Disease Control & Prevention, Atlanta, Georgia, USA; d Science and Technology Directorate, U.S. Department of Homeland Security, Washington, DC, USA; e Office of the Commissioner, U.S. Food & Drug Administration, Silver Spring, Maryland, USA; f Department of Pathology and Microbiology, Aga Khan University, Karachi, Pakistan

## Abstract

The emergence and spread of colistin resistance among multidrug-resistant (MDR) *Klebsiella pneumoniae* represent a critical threat to global health. Here, we report the complete genome sequences of 10 MDR, colistin-susceptible and -resistant *K. pneumoniae* clinical isolates obtained in Pakistan between 2010 and 2013.

## GENOME ANNOUNCEMENT

*Klebsiella pneumoniae* is a Gram-negative bacterial pathogen that causes a range of clinical diseases including pneumonia, bacteremia, and wound and urinary tract infections. The continuing increase of antibiotic resistance in *K. pneumoniae* presents a considerable challenge to global health. In particular, carbapenemase and/or extended spectrum β-lactamase production by this important human pathogen greatly limits therapeutic options and is associated with frequent treatment failures and increased mortality (1). The serious challenge posed by multidrug-resistant (MDR) *K. pneumoniae* has been countered by the clinical use of colistin (CST), a decades-old polymyxin considered to be the last line of defense against infections caused by MDR Gram-negative bacterial pathogens. Unfortunately, reports of CST-resistant *K. pneumoniae* are becoming increasingly common, and surveillance studies have demonstrated an increase in the prevalence of CST resistance (2). Infections caused by MDR, CST-resistant *K. pneumoniae* are of great concern as there are no suitable therapeutic agents available to treat them.

Here, we report the whole-genome sequences for 10 MDR *K. pneumoniae* strains isolated from patients in Pakistan between 2010 and 2013. Among these strains, seven are CST resistant and three are CST susceptible as determined using the Vitek 2 system (bioMérieux, Marcy l’Etoile, France) and by disk diffusion. These data provide a comparative genetic context for CST resistance in *K. pneumoniae* that will inform infectious diseases epidemiology and the identification of antimicrobial resistance determinants. Importantly, such information is likely to be broadly applicable to CST resistance among *Enterobacteriaceae*.

DNA libraries were prepared using the Nextera XT DNA library preparation kit (Illumina, San Diego, CA, USA), and whole-genome sequencing was performed on an Illumina MiSeq system using MiSeq reagent kit v2 (2 × 250 bp paired-end reads). *De novo* genome assemblies were created using SPAdes Genome Assembler and evaluated in comparison to the genome of *K. pneumoniae* HS11286 (3) using the quality assessment tool QUAST (4). Assembled genomes were annotated using the National Center for Biotechnology Information (NCBI) Prokaryotic Genome Annotation Pipeline (https://www.ncbi.nlm.nih.gov/genome/annotation_prok/). Multi-locus sequence typing (MLST) was performed according to the scheme described for the Institut Pasteur *K. pneumoniae* database (http://bigsdb.pasteur.fr/klebsiella/klebsiella.html). The presence of NDM-1 and/or OXA-48 β-lactamases was determined by PCR and confirmed using whole-genome sequencing data. The presence of NDM-1 and/or OXA-48, CST resistance, and sequence type (ST) are indicated for each strain: BA2880 (OXA-48, CST^R^, ST101), BA3783 (NDM-1 and OXA-48, CST^R^, ST14), BL13802 (NDM-1 and OXA-48, CST^S^, ST11), BA2664 (OXA-48, CST^R^, ST11), BL8800 (OXA-48, CST^R^, ST101), BL12456 (NDM-1, CST^S^, ST14), BL849 (NDM-1, CST^R^, ST11), BU19801 (NDM-1, CST^R^, ST307), MS84 (NDM-1, CST^R^, ST15), BL12125 (NDM-1, CST^S^, ST14).

### Accession number(s).

The genome assemblies described in this manuscript are available in DDBJ/ENBL/GenBank under the accession numbers MAGC00000000 (BA2880), MAGE00000000 (BA3783), MAGF00000000 (BL13802), MAGG00000000 (BA2664), MAGH00000000 (BL8800), MAGI00000000 (BL12456), MAGJ00000000 (BL849), MAGK00000000 (BU19801), MAGL00000000 (MS84), and MAGM00000000 (BL12125).
